# Supplementary material for: Dietary Iron Overload Differentially Modulates Chemically-Induced Liver Injury in Rats
Source: Nutrients. 2020 Sep 11;12(9):2784. doi: 10.3390/nu12092784 (PMC7551424; doi:10.3390/nu12092784)
Supplement: Supplementary file 1 [file nutrients-12-02784-s001.zip › nutrients-900462-supplementary.pptx]

## Slide 1
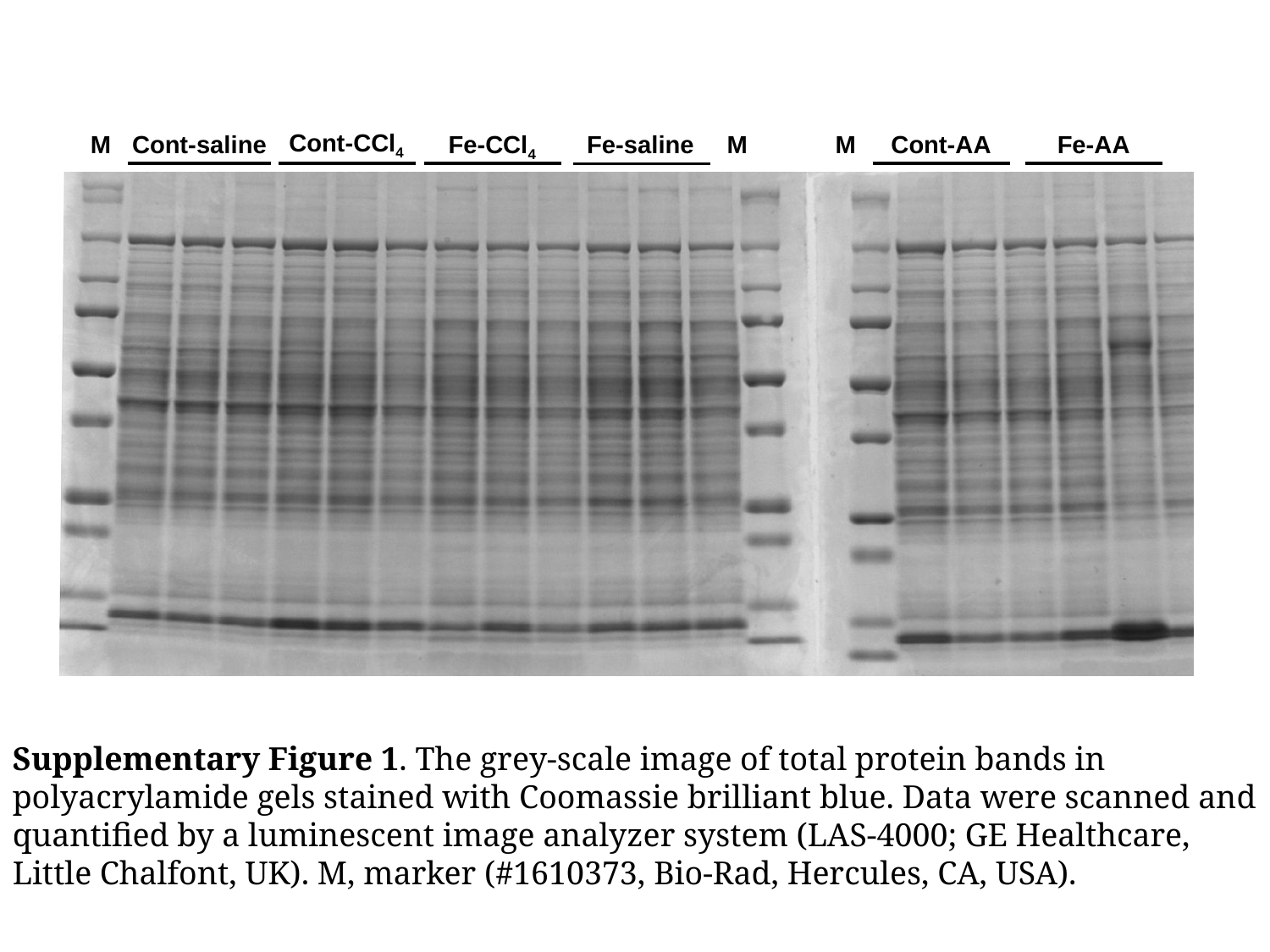

Cont-CCl4
M
M
M
Fe-AA
Cont-AA
Cont-saline
Fe-CCl4
Fe-saline
Supplementary Figure 1. The grey-scale image of total protein bands in polyacrylamide gels stained with Coomassie brilliant blue. Data were scanned and quantified by a luminescent image analyzer system (LAS-4000; GE Healthcare, Little Chalfont, UK). M, marker (#1610373, Bio-Rad, Hercules, CA, USA).

## Slide 2
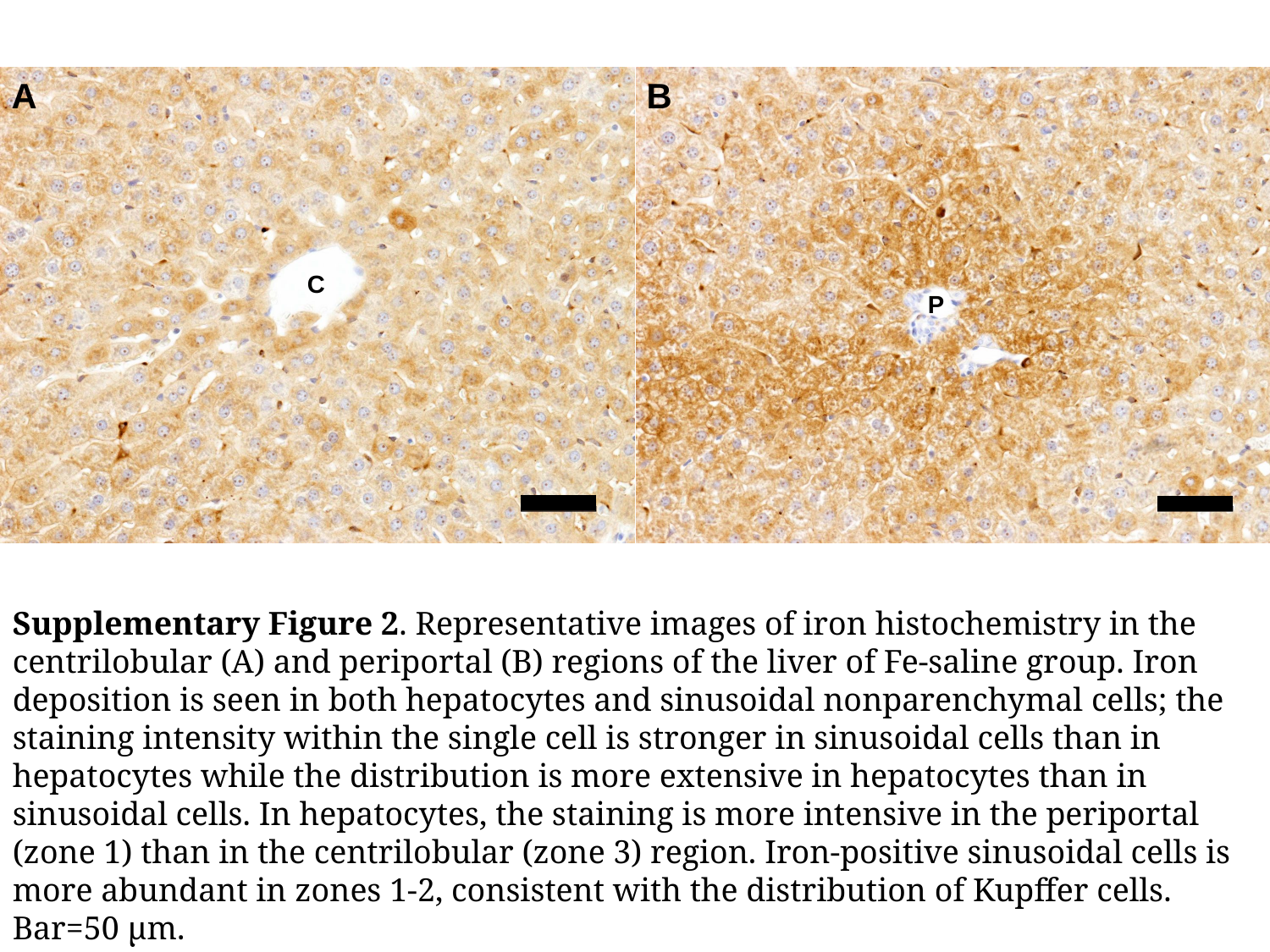

A
B
C
P
Supplementary Figure 2. Representative images of iron histochemistry in the centrilobular (A) and periportal (B) regions of the liver of Fe-saline group. Iron deposition is seen in both hepatocytes and sinusoidal nonparenchymal cells; the staining intensity within the single cell is stronger in sinusoidal cells than in hepatocytes while the distribution is more extensive in hepatocytes than in sinusoidal cells. In hepatocytes, the staining is more intensive in the periportal (zone 1) than in the centrilobular (zone 3) region. Iron-positive sinusoidal cells is more abundant in zones 1-2, consistent with the distribution of Kupffer cells. Bar=50 μm.

## Slide 3
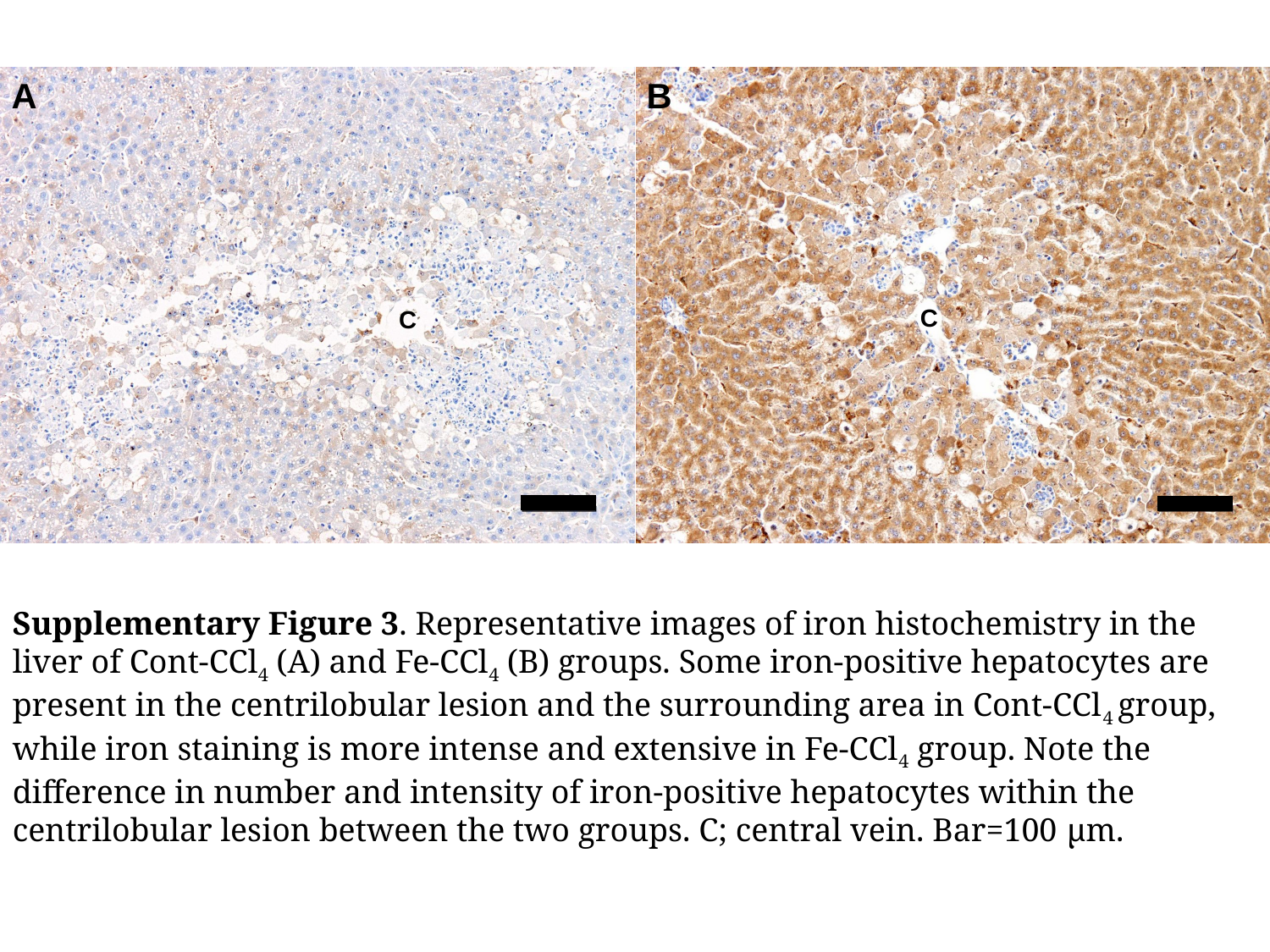

A
B
C
C
Supplementary Figure 3. Representative images of iron histochemistry in the liver of Cont-CCl4 (A) and Fe-CCl4 (B) groups. Some iron-positive hepatocytes are present in the centrilobular lesion and the surrounding area in Cont-CCl4 group, while iron staining is more intense and extensive in Fe-CCl4 group. Note the difference in number and intensity of iron-positive hepatocytes within the centrilobular lesion between the two groups. C; central vein. Bar=100 μm.

## Slide 4
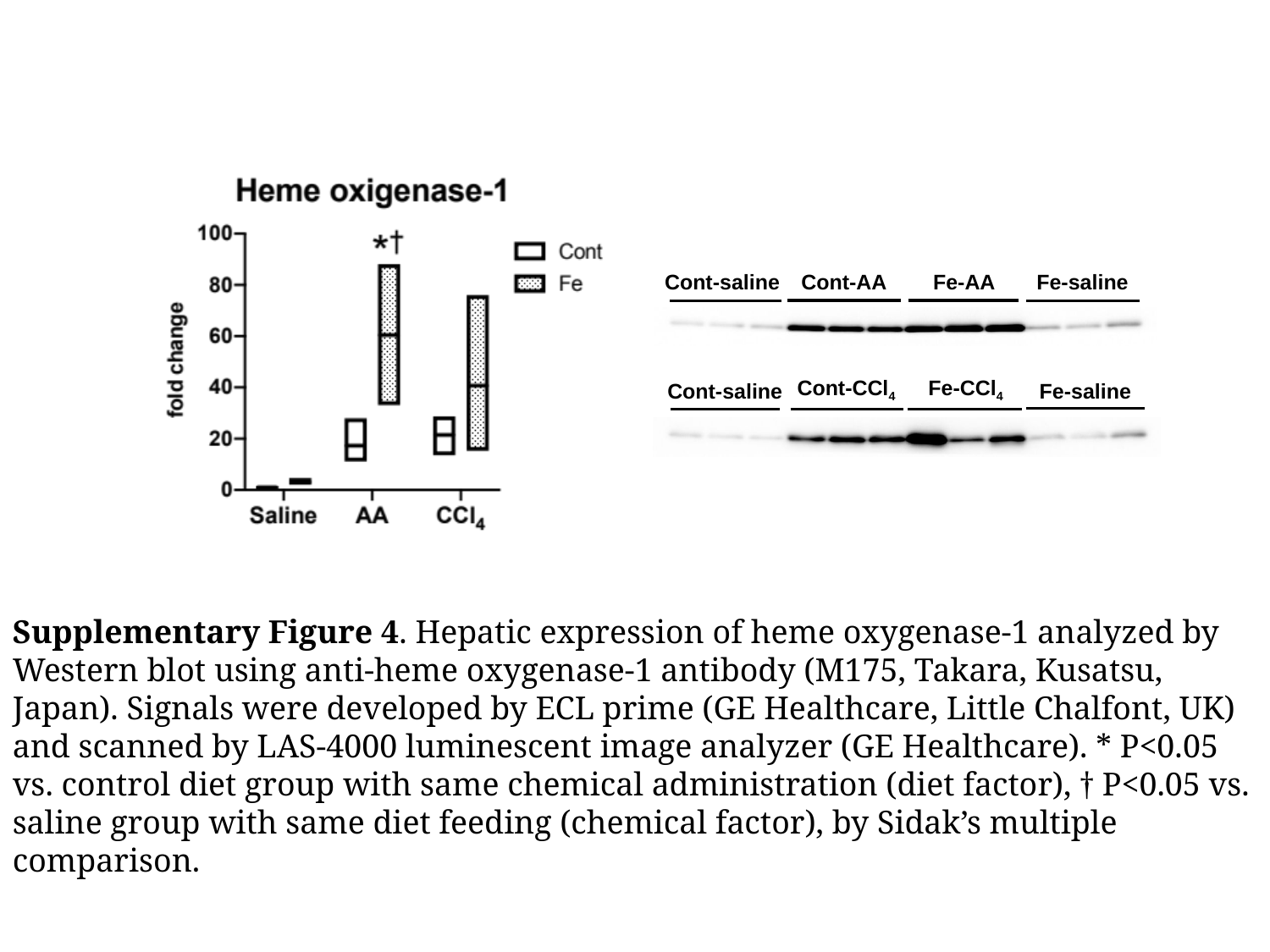

Fe-AA
Cont-AA
Fe-saline
Cont-saline
Cont-saline
Fe-CCl4
Fe-saline
Cont-CCl4
Supplementary Figure 4. Hepatic expression of heme oxygenase-1 analyzed by Western blot using anti-heme oxygenase-1 antibody (M175, Takara, Kusatsu, Japan). Signals were developed by ECL prime (GE Healthcare, Little Chalfont, UK) and scanned by LAS-4000 luminescent image analyzer (GE Healthcare). * P<0.05 vs. control diet group with same chemical administration (diet factor), † P<0.05 vs. saline group with same diet feeding (chemical factor), by Sidak’s multiple comparison.
